# Promiscuous methionyl-tRNA synthetase mediates adaptive mistranslation to protect cells against oxidative stress

**DOI:** 10.1242/jcs.152470

**Published:** 2014-10-01

**Authors:** Jin Young Lee, Dae Gyu Kim, Byung-Gyu Kim, Won Suk Yang, Jeena Hong, Taehee Kang, Young Sun Oh, Kyung Rok Kim, Byung Woo Han, Byung Joon Hwang, Beom Sik Kang, Mi-Sun Kang, Myung-Hee Kim, Nam Hoon Kwon, Sunghoon Kim

**Affiliations:** 1 Medicinal Bioconvergence Research Center, College of Pharmacy, Seoul National University, Seoul 151-742, Korea; 2 Research Institute of Pharmaceutical Sciences, Department of Pharmacy, College of Pharmacy, Seoul National University, Seoul 151-742, Korea; 3 Department of Molecular Bioscience, Kang Won National University, Chuncheon-si, Gangwon-do 200-701, Korea; 4 School of Life Science and Biotechnology, Kyungpook National University, Daegu 702-701, Korea; 5 Department of Computer Science and Engineering Center for Computer Graphics and Virtual Reality, Ewha Womans University, Seoul 120-750, Korea; 6 WCU Department of Molecular Medicine and Biopharmaceutical Sciences, Graduate School of Convergence Science and Technology, Seoul National University, Suwon 443-270, Korea

**Keywords:** Cell protection, ERK, Methionyl-tRNA synthetase, Misacylation, Reactive oxygen species

## Abstract

Aminoacyl-tRNA synthetases (ARSs) acylate transfer (t)RNAs with amino acids. Charging tRNAs with the right amino acids is the first step in translation; therefore, the accurate and error-free functioning of ARSs is an essential prerequisite for translational fidelity. A recent study found that methionine (Met) can be incorporated into non-Met residues of proteins through methionylation of non-cognate tRNAs under conditions of oxidative stress. However, it was not understood how this mis-methionylation is achieved. Here, we report that methionyl-tRNA synthetase (MRS) is phosphorylated at Ser209 and Ser825 by extracellular signal-related kinase (ERK1/2) under conditions of stress caused by reactive oxygen species (ROS), and that this phosphorylated MRS shows increased affinity for non-cognate tRNAs with lower affinity for tRNA^Met^, leading to an increase in Met residues in cellular proteins. The expression of a mutant MRS containing the substitutions S209D and S825D, mimicking dual phosphorylation, reduced ROS levels and cell death. This controlled inaccuracy of MRS seems to serve as a defense mechanism against ROS-mediated damage at the cost of translational fidelity.

## INTRODUCTION

Reactive oxygen species (ROS), which are generally unstable and highly reactive, are continuously produced because of normal physiological events such as aerobic metabolism as well as extracellular stress, including that resulting from exposure to radiation or chemicals. Low levels of ROS promote cellular proliferation and differentiation, but long-term or high-level exposure to ROS induces oxidative damage, causing cell death. Maintaining redox homeostasis is therefore essential for normal cell growth and survival (Trachootham et al., 2009). By contrast, an ROS imbalance is related to several pathophysiological conditions, including cancer, diabetes, chronic inflammation, atherosclerosis, ischemia-reperfusion injury and neurodegenerative disorders (Jones et al., 2012; Waris and Ahsan, 2006).

To protect against oxidative damage, cells make use of various endogenous enzymatic and non-enzymatic antioxidants, including peroxidase, superoxide dismutase, glutathione, NADPH, ubiquinone, vitamins and carotenoids (Birben et al., 2012). Apart from these antioxidant molecules, a distinct antioxidant mechanism uses methionine (Met) as a ROS scavenger to protect proteins (Luo and Levine, 2009). Met residues on the surface of proteins intercept ROS such as hydrogen peroxide, and the residues are converted to methionine sulfoxides. The oxidized Met is then reduced to Met by methionine sulfoxide reductases (Levine et al., 1996; Levine et al., 2000; Luo and Levine, 2009; Stadtman et al., 2002; Stadtman et al., 2003).

It was recently shown that methionylation to non-cognate tRNAs, termed ‘Met-misacylation’, increased by up to 10% in mammalian cells under oxidative stress (Netzer et al., 2009; Wiltrout et al., 2012). This Met-misacylation predominantly occurs in tRNA families that originally carry charged or polar amino acids (Netzer et al., 2009), implying that methionyl-tRNA synthetase (MRS) might be involved in Met-misacylation to non-cognate tRNAs such as tRNA^Lys^, tRNA^Gly^ and tRNA^Leu^ (Netzer et al., 2009). However, the molecular mechanism responsible for Met-misacylation under oxidative stress remains unidentified.

MRS is an enzyme that is responsible for the ligation of Met to the cognate initiator or elongator tRNA^Met^. In the mammalian system, MRS is normally bound to the multi-tRNA synthetase complex (MSC), making specific interaction with aminoacyl-tRNA synthetase (ARS)-interacting multifunctional protein 3 (AIMP3, also known as EEF1E1 or p18) (Park et al., 2005). Our recent study suggests that the phosphorylation of MRS reduces its affinity for the cognate tRNA, resulting in suppression of global translation (Kwon et al., 2011). This study suggested that the tRNA binding affinity of MRS can be modulated by post-translational modification. Further expanding this discovery, we hypothesized that certain modifications of MRS could alter its tRNA specificity and induce Met-misacylation to non-cognate tRNAs, which would serve as a protective mechanism against oxidative stress. In this study, we investigated the functional significance of MRS in protection against ROS damage and in the molecular mechanism that controls the specificity of tRNA recognition.

## RESULTS

### MRS is phosphorylated by ERK during oxidative stress

**Fig. 1.**
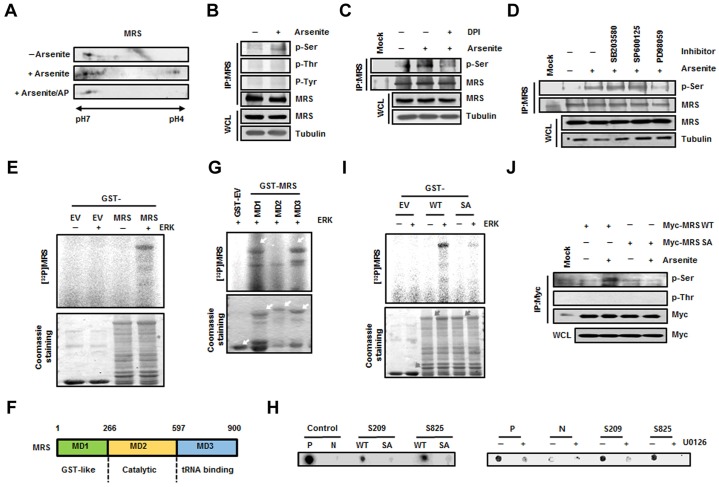
**Determination of ERK-mediated phosphorylation sites in MRS during ROS stress.** (A) Lysates from untreated and sodium-arsenite-treated HeLa cells were subjected to 2D-PAGE. The gel was immunoblotted with an anti-MRS antibody. To check ROS-dependent phosphorylation of MRS, lysates from sodium-arsenite-treated cells were incubated with alkaline phosphatase (AP). (B) Lysates prepared as above were immunoprecipitated (IP) with the anti-MRS antibody and immunoblotted with antibodies against phosphoserine (p-Ser), phosphothreonine (p-Thr) and phosphotyrosine (p-Tyr). WCL, whole-cell lysate. (C) Lysates from HeLa cells treated with sodium arsenite with or without DPI (ROS inhibitor) were immunoprecipitated using the anti-MRS antibody. The gel was immunoblotted with antibody against p-Ser. (D) HeLa cells pre-treated with each MAPK inhibitor – SB203580 (p38 MAPK inhibitor), SP600125 (JNK inhibitor) and PD98059 (ERK inhibitor) – were incubated in medium containing sodium arsenite. The lysates were immunoprecipitated with the anti-MRS antibody and immunoblotted with antibody against p-Ser. (E) Purified GST (EV, empty vector) and GST–MRS were subjected to an *in vitro* kinase reaction by incubating with ERK and [γ-^32^P]ATP. After staining with Coomassie Brilliant Blue, radioactivity was detected by autoradiography. (F) Schematic representation of functional domains in human MRS. The domains of MRS can be divided into MD1 (GST-like, residues 1–266), MD2 (Catalytic, residues 267–597) and MD3 (tRNA-binding, residues 598–900) fragments. (G) Individual GST-fused domains of MRS were subjected to an *in vitro* kinase assay with ERK in the presence of [γ-^32^P]ATP. Phosphorylation signal was detected by autoradiography. (H) HEK293T cells transfected with GFP–ERK were immunoprecipitated with anti-GFP antibody. The immunoprecipitated GFP–ERK was mixed with each biotinylated synthetic peptide (WT, wild type; SA, phosphorylation-inactive form) and [γ-^32^P]ATP. A consensus sequence phosphorylated by ERK was used as a control (P, positive control; N, phosphorylation-inactive form of positive control) (left). Positive control peptide and peptides containing each S209 and S825 were incubated with [γ-^32^P]ATP and immunoprecipitated ERK from cells pre-treated with U0126. Autoradioactivity from the peptides was detected after dot blotting (right). (I) To confirm the ERK-dependent phosphorylation sites in MRS, an *in vitro* kinase assay was performed with wild-type GST–MRS and the GST–MRS-S209A/S825A (SA) mutant as described above. The phosphorylation signal was detected by autoradiography. The relative quantification obtained by densitometric analysis for the phosphorylation signal of wild-type MRS and MRS-SA was 1 and 0.11, respectively. (J) HEK293T cells transfected with wild-type Myc–MRS or the Myc–MRS-SA mutant were treated with sodium arsenite and immunoprecipitated using the anti-Myc antibody. Arsenite-dependent phosphorylation of MRS was detected using the antibody against p-Ser.

Based on previous studies showing the possibility of MRS-mediated Met-misacylation (Jones et al., 2011; Netzer et al., 2009; Wiltrout et al., 2012) and modulation of MRS catalytic activity by phosphorylation (Kwon et al., 2011; Pendergast and Traugh, 1985), we first examined MRS modification under ROS stress. Protein extracts from HeLa cells treated with arsenite, a ROS-inducing agent, were separated by two-dimensional polyacrylamide gel electrophoresis (2D-PAGE) and then immunoblotted using the anti-MRS antibody. Additional spots of MRS were present on the acidic side following treatment with arsenite; these spots disappeared after alkaline phosphatase treatment, indicating that MRS was phosphorylated under ROS stress (Fig. 1A). To identify which amino acid residues were involved in ROS-mediated phosphorylation, proteins from control and ROS-induced HeLa cells were immunoprecipitated with an anti-MRS antibody and immunoblotted with phospho-specific antibodies. Phosphorylation of MRS at serine residues was observed in MRS extracted from arsenite-treated cells (Fig. 1B), whereas phosphorylation at threonine or tyrosine residues was not detected. Serine-specific phosphorylation was also confirmed in H_2_O_2_-treated HeLa cells (supplementary material Fig. S1A). Removal of ROS by treatment with diphenyleneiodonium (DPI), a broad inhibitor of oxidases, apparently reduced the arsenite-dependent phosphorylation of MRS (Fig. 1C), indicating that kinases activated under ROS signaling are involved in MRS phosphorylation. Because mitogen-activated protein kinases (MAPKs) are well known for their various functions upon stimulation by ROS (Lau et al., 2004; Pan et al., 2009; Son et al., 2011), we treated HeLa cells with MAPK-specific inhibitors and monitored MRS phosphorylation. Serine-specific phosphorylation of MRS induced by arsenite was dramatically suppressed in cells treated with the ERK inhibitor PD98059, whereas p38 MAPK (MAPK11–MAPK14) or c-Jun N-terminal kinase (JNK, MAPK8–MAPK10) inhibitors did not influence MRS phosphorylation (Fig. 1D). Next, we performed an *in vitro* kinase assay by incubating glutathione sulfotransferase (GST) or GST–MRS with purified active ERK1/2 (referred to here as ERK) and [γ-^32^P]ATP to confirm whether MRS is a real substrate for ERK. GST–MRS, but not GST, showed obvious phosphorylation signal when incubated with ERK (Fig. 1E); therefore, we concluded that MRS was phosphorylated at serine residues by ERK under ROS stress.

### Determination of the ERK-induced phosphorylation sites in MRS

Human MRS consists of three functional domains, the GST-like (MD1, residues 1–266), catalytic (MD2, residues 267–597) and tRNA-binding (MD3, residues 598–900) domains (Fig. 1F). Using these domain fragments, we conducted an *in vitro* kinase assay to determine which domain of MRS undergoes ERK-mediated phosphorylation. Because a strong phosphorylation signal was observed in MD1 and MD3, but not in MD2 (Fig. 1G), we analyzed phosphorylation sites in MD1 and MD3 after the *in vitro* kinase assay by mass spectrometry to determine the ERK-dependent phosphorylation sites in MRS. Among the phosphorylation sites of MRS detected (supplementary material Fig. S2A), we selected the serine residues Ser209 and Ser825, because ROS-induced MRS phosphorylation is serine-specific (Fig. 1B). We synthesized biotinylated MRS peptides containing Ser209 and Ser825, as well as the same peptides with serine to alanine substitutions. The peptide kinase assay revealed the apparent phosphorylation of both Ser209- and Ser825-containing peptides by ERK, whereas little signal was observed in alanine-substituted mutant peptides (Fig. 1H, left) or under ERK inhibitor-treated conditions (Fig. 1H, right). The same results were obtained when the *in vitro* kinase assay was performed with wild-type and mutant GST–MRS proteins. The GST–MRS-S209A/S825A (SA) mutant, in which both serine residues were replaced with alanines, showed minimal phosphorylation upon incubation with ERK compared with wild-type MRS (Fig. 1I). We also transfected HEK293T cells with wild-type Myc–MRS or the Myc–MRS-SA mutant and analyzed serine-specific phosphorylation by immunoblotting. The phosphorylation signal was increased in wild-type MRS by arsenite treatment, but was not detected in the dual-alanine-substituted mutant (Fig. 1J). Moreover, H_2_O_2_ treatment did not induce phosphorylation in the MRS-SA mutant (supplementary material Fig. S1A). We also checked the phosphorylation state in single-alanine-substituted mutants. Although the serine-specific phosphorylation signal was slightly lower in the S209A and S825A single mutants compared with that of wild-type MRS, these single-alanine-substituted mutants did not show a dramatic decrease as seen with the MRS-SA mutant, suggesting that the Ser209 and Ser825 residues are dually phosphorylated by ERK during ROS stress (supplementary material Fig. S2B).

ERK is activated in response to various stimuli including UV, therefore we wondered whether Ser209 and Ser825 phosphorylation is specific to ROS. We transfected Myc–MRS-S662A into HEK293T cells along with wild-type Myc–MRS and investigated MRS phosphorylation. The Ser662 residue of MRS is known to be phosphorylated by general control nonderepressible 2 (GCN2) upon UV irradiation (Kwon et al., 2011). If Ser209 or Ser825 can be phosphorylated by UV-activated ERK, the phosphorylation signal would be detected in MRS-S662A following UV treatment. Phosphorylation of MRS-S662A, however, was only detected under ROS stress but not in response to UV, suggesting that Ser209 and Ser825 phosphorylation is specific to ROS stress (supplementary material Fig. S2C).

### Phosphorylation of MRS at Ser209 and Ser825 induces Met-misacylation under ROS stress

**Fig. 2.**
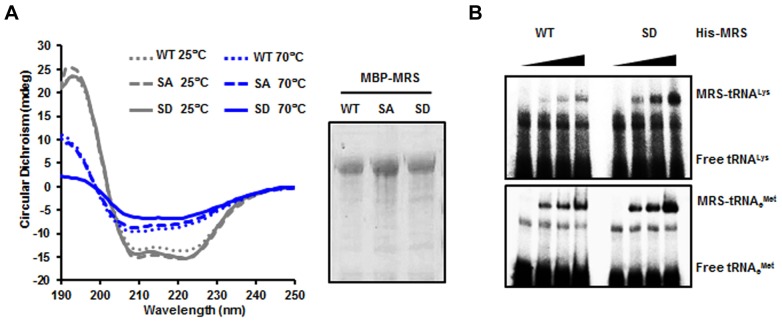
**Dually phosphorylated MRS undergoes a conformational change and binds to tRNA^Lys^(CUU).** (A) Maltose-binding protein (MBP)-tagged wild-type (WT) MRS and the MBP–MRS-SA and SD mutants were purified, and the circular dichroism spectra of these proteins were obtained in the far-UV at two different temperatures (left). The purified proteins were separated by using SDS-PAGE and stained with Coomassie Brilliant Blue (right). (B) Binding affinities of wild-type MRS and the S209D/S825D (SD) mutant to tRNA^Lys^(CUU) (upper panel) and tRNAe^Met^(CAU) (lower panel) were determined by EMSA. Each tRNA probe was incubated with wild-type His–MRS and His–MRS-SD proteins and separated by non-denaturing PAGE.

Because MRS was modified by phosphorylation during ROS stress, we investigated the correlation between Met-misacylation and the dual phosphorylation of MRS under ROS stress conditions. We first analyzed circular dichroism spectra of wild-type MRS, the MRS-SA mutant and the S209D/S825D (SD) mutant and observed a temperature-dependent structural change of the MRS-SD mutant in the far-UV spectra (Fig. 2A). To evaluate the Met-misacylation ability of the MRS-SD mutant, we performed *in vitro* tRNA binding and aminoacylation activity assays. Based on a previous report suggesting that ROS-dependent Met-misacylation predominantly occurs in tRNA^Lys^(CUU) (Netzer et al., 2009), we tested tRNA^Lys^(CUU) first. We examined the interaction between the MRS-SD mutant and tRNA^Lys^(CUU) by using an electrophoretic mobility shift assay (EMSA). The MRS-SD mutant showed a clear increase in association with radioactively labeled tRNA^Lys^(CUU) in a dose-dependent manner, whereas wild-type MRS did not (Fig. 2B, upper panel). Both wild-type MRS and the MRS-SD mutant interacted with elongator tRNA^Met^ (tRNAe^Met^)(CAU) to a similar extent (Fig. 2B, lower panel).

In the aminoacylation activity assay, the K_M_ value of the MRS-SD mutant for tRNA^Lys^(CUU) was about sixfold higher than that of wild-type MRS for tRNAe^Met^(CAU) (Table 1). It is notable that the MRS-SD mutant showed relatively higher affinity for tRNA^Lys^(CUU) than for tRNAe^Met^(CAU), whereas the K_M_ of wild-type MRS for tRNA^Lys^(CUU) was not measurable. Although the MRS-SA mutant showed a significant decrease in its catalytic activity against tRNA^Met^, it retained its specificity to the cognate tRNA. We further confirmed the Met-misacylation ability of the MRS-SD mutant to other types of tRNA, such as tRNA^Ala^(AGC), tRNA^Gly^(GCC), tRNA^His^(GUG) and tRNA^Leu^(CAG). The MRS-SD mutant generally showed a considerable increase in binding affinities to these non-cognate tRNAs compared with that of wild-type MRS (supplementary material Fig. S3A). Consistently, the MRS-SD mutant charged non-cognate tRNAs with Met at levels similar to those seen with tRNAe^Met^(CAU), with the highest activity for tRNA^Lys^(CUU) among the tRNAs (supplementary material Fig. S3B). These results suggested that dual phosphorylation of MRS at residues Ser209 and Ser825 can endow MRS with the ability to recognize non-cognate tRNAs and misacylate them with Met.

**Table 1.**
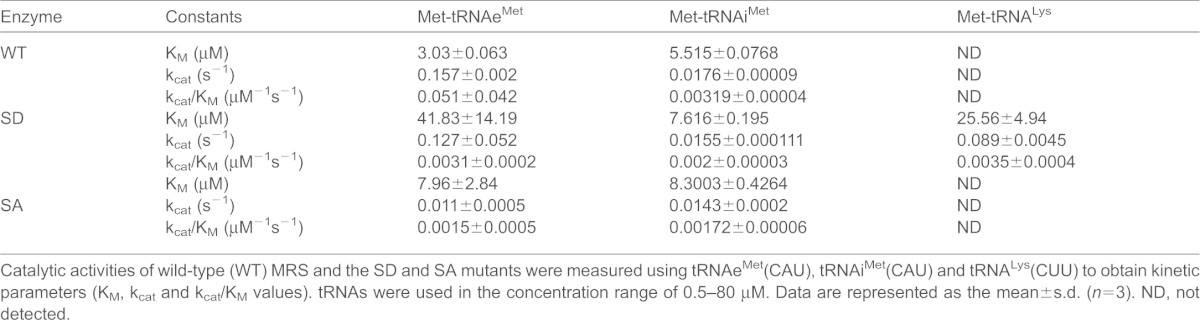
Comparison of catalytic activities between wild-type MRS, MRS-SA and MRS-SD

Catalytic activities of wild-type (WT) MRS and the SD and SA mutants were measured using tRNAe^Met^(CAU), tRNAi^Met^(CAU) and tRNA^Lys^(CUU) to obtain kinetic parameters (K_M_, k_cat_ and k_cat_/K_M_ values). tRNAs were used in the concentration range of 0.5–80 µM. Data are represented as the mean±s.d. (*n* = 3). ND, not detected.

**Fig. 3.**
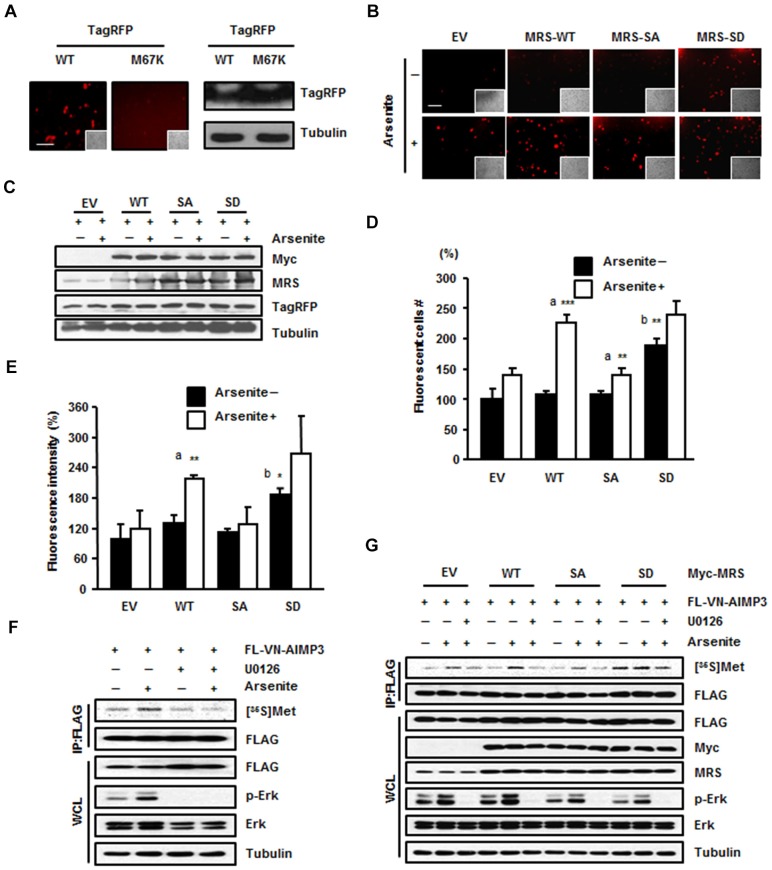
**Dually phosphorylated MRS induces mismethionylation under ROS stress.** (A) HEK293T cells were transfected with wild-type (WT) TagRFP or the TagRFP M67K mutant and the fluorescence of each protein was compared (left). Insets show the same field as in the phase-contrast image. Scale bar: 80 µm. The expression level of wild-type TagRFP and the TagRFP M67K mutant were determined by immunoblotting (right). (B–E) MRS-dependent Met-misincorporation was monitored using the TagRFP M67K mutant, the fluorescence of which disappeared due to the M to K substitution. HEK293T cells co-transfected with Tag-RFP M67K and empty vector (EV) or each type of Myc–MRS (WT, SA or SD mutant) were treated with sodium arsenite. Revival of fluorescence due to Met-misincorporation at the M67K residue position was observed by fluorescence microscopy (×200). Insets show the same field as in the phase-contrast images. Scale bar: 80 µm (B). Expression levels of total MRS, Myc–MRS (wild type, SA or SD mutant) and TagRFP M67K mutant were analyzed by immunoblotting (C). The relative number (D) and the relative fluorescence intensity (E) of red fluorescent cells are presented as bar graphs. Data are represented as the mean±s.d. (*n* = 3); *P<0.05; **P<0.01; ***P<0.001; a, *P*-value indicates a significant difference between the arsenite-untreated and -treated groups; b, *P*-value indicates a significant difference between arsenite-untreated empty vector and SD groups. (F) HEK293T cells transfected with FLAG-VN–AIMP3 were incubated with arsenite, [^35^S]Met and with or without the ERK inhibitor U0126. The radioactive signal from the immunoprecipitated (IP) FLAG-VN–AIMP3 was detected by autoradiography. WCL, whole-cell lysate. (G) HEK 293T cells co-transfected with FLAG-VN–AIMP3 and empty vector or each type of Myc–MRS (wild type, SA or SD mutant) were incubated with [^35^S]Met in the presence with arsenite. To see the effect of ERK inhibitor, cells were pre-treated with U0126 1 h before the arsenite treatment. The cell extracts were immunoprecipitated with anti-FLAG antibody. [^35^S]Met signals from the FLAG-VN–AIMP3 were monitored by autoradiography.

Next, we investigated whether Met-misacylated tRNA families can be used for translation. Because MRS incorporation into the MSC might affect translational efficiency, we firstly performed an immunoprecipitation assay using anti-EPRS (glutamyl-prolyl tRNA synthetase) and anti-Myc antibodies to confirm the presence of the Myc–MRS-SD mutant in the MSC. EPRS, the representative subunit of the MSC, and the Myc–MRS-SD mutant were co-immunoprecipitated together; therefore, we concluded that exogenous MRS-SD mutant can localize to the MSC like endogenous MRS (data not shown). We set up a fluorescence system using TagRFP to monitor Met-misacylation. TagRFP is a red fluorescent protein in which the Met67 residue is crucial for fluorescence. We mutated the ATG codon for Met67 to AAG for Lys to turn off the fluorescence and observed that the red fluorescence signal from the TagRFP-M67K mutant was undetectable (Fig. 3A). If MRS mismethionylated tRNA^Lys^(CUU), then Met-tRNA^Lys^(CUU) could be incorporated into the mutated codon of TagRFP-M67K to restore the Lys residue to original Met, thereby restoring red fluorescence. We transfected the TagRFP-M67K mutant together with empty vector, wild-type Myc–MRS, the Myc–MRS-SA mutant or the Myc–MRS-SD mutant into HEK293T cells. Empty-vector-, wild-type-MRS- and MRS-SA-expressing cells exhibited increased red fluorescent signal upon arsenite treatment, with the most enhanced fluorescence in wild-type-MRS-expressing cells (Fig. 3B). The MRS-SD mutant showed increased fluorescent signal regardless of arsenite treatment (Fig. 3B). Based on the results of immunoblotting, this difference in fluorescence among the MRS proteins was not due to the expression level of TagRFP (Fig. 3C). We analyzed the images based on the number of fluorescence-positive cells (Fig. 3D) as well as fluorescence intensity (Fig. 3E). Both of these sets of results revealed three notable points: (1) the occurrence of ROS-responsive Met-misincorporation by endogenous and exogenous wild-type MRS; (2) a similar increase in Met-misincorporation under ROS conditions in the cells expressing empty vector or the MRS-SA mutant; and (3) basally enhanced Met-misincorporation by the MRS-SD mutant. Similar patterns were also observed with the TagRFP-M67H and TagRFP-M67G mutants, indicative of the MRS-mediated production of Met-tRNA^His^(GUG) and Met-tRNA^Gly^(UCC), respectively, and their incorporation into the TagRFP mutant protein (supplementary material Fig. S3C).

**Fig. 4.**
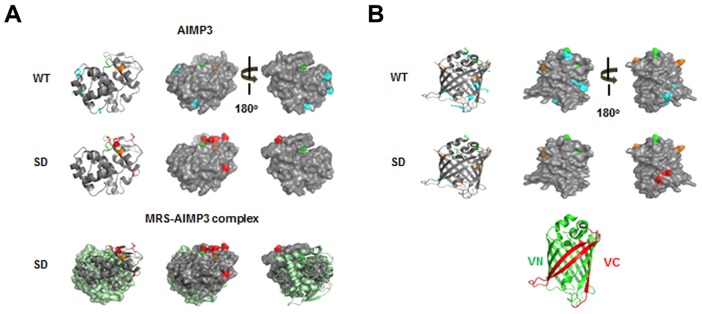
**Met-misincorporated residues in Flag-VN–AIMP3 and their location in the AIMP3 and Venus structures.** (A,B) The Met-exchanged residues identified by mass spectrometry analysis are depicted in the AIMP3 (pdb2uz8) (A) and the full Venus (pdb3t6h) (B) structures. Residues only identified from HEK293T cells expressing wild-type (WT) MRS (A,B, upper images) and MRS-SD (A,B, middle images) are depicted in cyan and red, respectively. Common residues identified from both cells are represented in orange. The original Met residue in AIMP3 is depicted in green. Mismethionylated residues on AIMP3 in MRS-SD-overexpressing conditions are also represented on the MRS–AIMP3 complex structure (pdb4bl7) (A, lower images). The GST-domain of MRS is shown in light green. Each N-terminus of Venus (VN) and deleted C-terminus of Venus (VC) in the full Venus protein structure is shown in green and red, respectively, for convenience (B, lower panel).

To further investigate the possible residue positions of a protein at which Met can be misincorporated, we first transfected HEK293T cells with the pBiFC-VN173-AIMP3 vector (Kwon et al., 2011) to allow us to use the FLAG-VN (N-terminal fragment of Venus)–AIMP3 protein as a reporter for monitoring Met-misincorporation. We chose FLAG-VN–AIMP3 because this protein is small (347 net amino acids without linker) and contains a relatively small number of Met residues (four amino acids). FLAG-VN–AIMP3 was immunoprecipitated after the addition of [^35^S]Met to the cells, and the autoradioactivity emanating from the FLAG-VN–AIMP3 was detected (Fig. 3F,G). The radioactive signal from FLAG-VN–AIMP3 was increased upon arsenite treatment, whereas the enhanced signal disappeared upon treatment with ERK inhibitor (Fig. 3F). This signal was not observed by serum stimulation either, which is another activation signal for ERK, suggesting that Met-misincorporation is a phenomenon that specifically occurs upon ROS stress (data not shown). We also confirmed MRS-dependent [^35^S]Met incorporation into FLAG-VN–AIMP3 in HEK293T cells expressing empty vector, wild-type MRS, the MRS-SA mutant or MRS-SD mutant. Autoradioactivity was detected in the protein isolated from arsenite-treated HEK293T cells with different signal intensity according to the expressional and mutational status of MRS. Wild-type-MRS-transfected cells showed the greatest signal intensity of ROS-dependent Met-misincorporation among the empty-vector-, wild-type-MRS- and the MRS-SA-expressing cells (Fig. 3G). The MRS-SD mutant induced the production of radioactive FLAG-VN–AIMP3 regardless of arsenite or ERK inhibitor treatment (Fig. 3G). This Met-misincorporation under ROS stress was MRS specific, because leucine-misincorporation into FLAG-VN–AIMP3 was not observed following overexpression of leucyl-tRNA synthetase (LRS) (supplementary material Fig. S3D). To further validate the MRS-mediated Met-misincorporation, we immunoprecipitated FLAG-VN–AIMP3 from HEK293T cell lysates and analyzed the Met-misincorporated residues using mass spectrometry. We identified several peptides containing Met at non-Met residue positions in the MRS-SD-expressing cells, although there was basal Met-misacylation in wild-type HEK293T cells (Table 2; Fig. 4; supplementary material Fig. S4). Interestingly, residues swapped with Met were detected on the surface of AIMP3 as well as Venus (Fig. 4A,B). In addition, the swapped residues of AIMP3 do not participate in the MRS interaction, and they are expected to be exposed outside of the MSC (Fig. 4A, lower panel). This supports the idea that Met-misincorporation is not an accidental event, but it results in the positioning of Met on the surface of proteins, allowing it to act as a ROS scavenger.

**Table 2.**
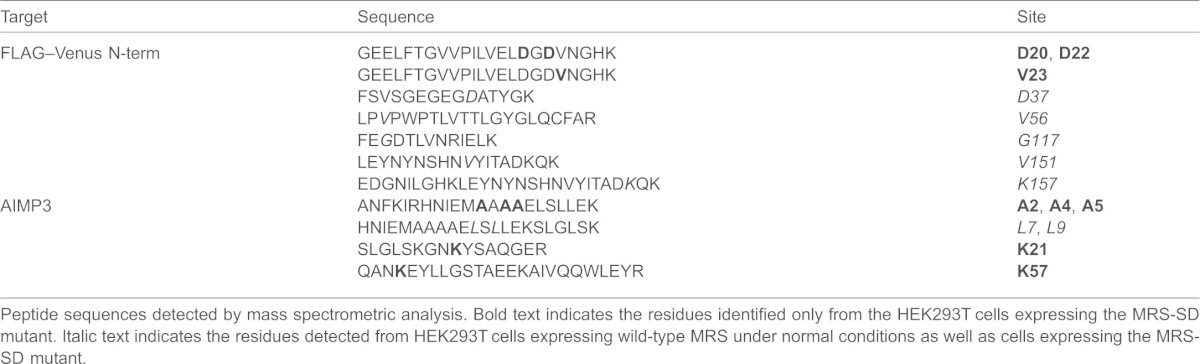
Met-misincorporated residues in Flag-VN–AIMP3

Peptide sequences detected by mass spectrometric analysis. Bold text indicates the residues identified only from the HEK293T cells expressing the MRS-SD mutant. Italic text indicates the residues detected from HEK293T cells expressing wild-type MRS under normal conditions as well as cells expressing the MRS-SD mutant.

### MRS-mediated Met-misacylation reduces intracellular ROS levels and protects cells from oxidative damage

**Fig. 5.**
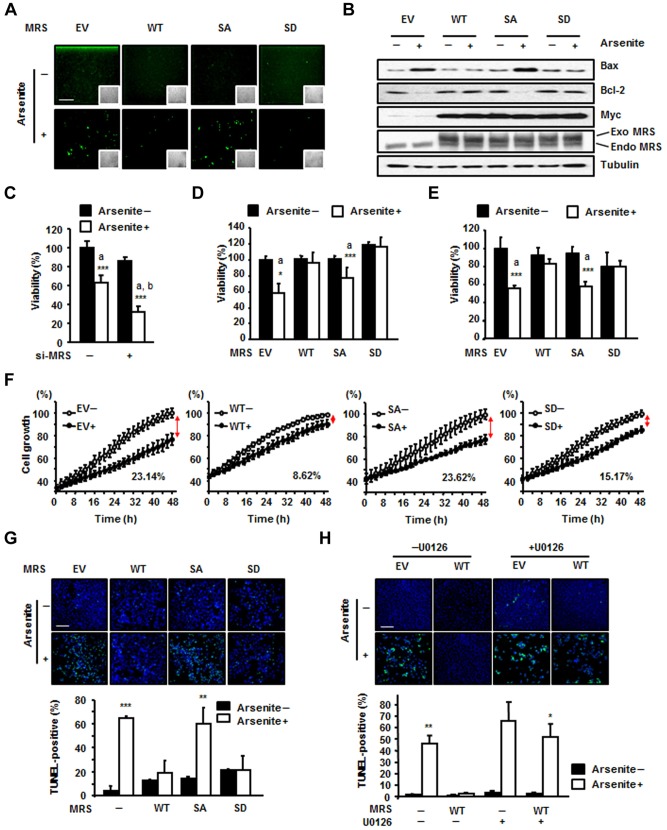
**Dually phosphorylated MRS reduces intracellular ROS levels and promotes cell survival under ROS stress.** (A) HEK293T cells were transfected with empty vector (EV) or Myc-tagged wild-type (WT) MRS, or the SA or SD mutants, and incubated with arsenite. ROS levels were detected by the DCFH-DA assay. Insets show the same field as in the phase-contrast images. Scale bar: 200 µm. (B) Bax and Bcl-2 levels were detected with their specific antibodies under the same conditions as shown in A. Exogenous (Exo) and endogenous (Endo) MRS were separated and detected using the anti-MRS antibody to show the expression level. (C) MRS level in HEK293T cells was reduced by treatment with MRS-specific siRNA (si-MRS) for 72 h. The effect of MRS expression on cell viability under ROS stress was determined by the MTT assay. (D,E) The effect of MRS proteins on cell viability under ROS stress was determined by the MTT assay. MRS proteins were transiently expressed in HEK293T cells (D) and stably expressed in HeLa cells (E). In C–E: a, *P*-value indicates a significant difference between the arsenite-untreated and -treated groups; b, *P*-value indicates a significant difference between arsenite-treated si-control and si-MRS groups. (F) The growth curves of MRS-expressing stably transfected HeLa cells were monitored in the presence and absence of ROS stress. (G) ROS-dependent apoptosis was determined in MRS-expressing stably transfected HeLa cells using the TUNEL assay. Cells incubated with or without arsenite for 72 h were fixed and stained with 4′,6-diamidino-2-phenylindole (DAPI; blue) and fluorescein-labeled dUTP. Green fluorescence indicates TUNEL-positive cells. Scale bar: 200 µm (upper panel). The number of TUNEL-positive cells was normalized to that of DAPI-positive cells, and the quantitative analysis is shown (lower panel). (H) HeLa cells expressing wild-type MRS were subjected to TUNEL assay as described for G with or without U0126 pre-treatment, and the images (upper panel) and the quantitative analysis (lower panel) for positive cells are shown. Scale bar: 200 µm. All quantitative data show the mean±s.d. (*n* = 3);*P<0.05; **P<0.01; ***P<0.001.

Because residue switching with Met was detected on the protein surface where ROS attack vulnerable amino acids, we examined whether MRS-mediated Met-misacylation can actually reduce intracellular ROS levels by using the dichloro-dihydro-fluorescein diacetate (DCFH-DA) assay. Whereas empty-vector-expressing control cells or MRS-SA-expressing cells showed slightly increased ROS levels upon arsenite treatment, wild-type-MRS- and MRS-SD-expressing cells did not (Fig. 5A). It is known that ROS increases the expression of pro-apoptotic Bax while reducing the expression level of Bcl-2, the Bax inhibitor (Chen et al., 1998; Fleury et al., 2002; Hossain et al., 2000). Thus, we investigated the expression levels of these markers and found that cells expressing wild-type MRS and the MRS-SD mutant were more resistant to apoptotic cell death upon arsenite-induced ROS generation than empty-vector- or MRS-SA-expressing cells (Fig. 5B). Taken together, these results suggest that MRS phosphorylation at Ser209 and Ser825 can reduce intracellular ROS levels, resulting in cell protection from ROS-mediated apoptosis.

To investigate the effect of MRS-mediated Met-misacylation on cell survival, we performed the 3-(4,5-dimethylthiazol-2-yl)-2,5-diphenyltetrazolium bromide (MTT) assay. First, we reduced the expression level of MRS using siRNA treatment. MRS knockdown significantly reduced the cell viability under ROS stress, suggesting the crucial role of MRS in cell protection against ROS stress (Fig. 5C). Overexpression of wild-type MRS or the MRS-SD mutant under ROS stress, whether transient (Fig. 5D) or stable (Fig. 5E), maintained cell survival at levels similar to that of normal growth in the MTT assay. By contrast, the MRS-SA mutant could not protect cells from oxidative damage as much as wild-type MRS or the MRS-SD mutant did, resulting in a significant reduction in cell viability, as shown by ROS-treated control cells. Growth curve analysis also showed the same results as the MTT assay. ROS retarded the growth of stably transfected HeLa cells expressing empty vector and the MRS-SA mutant by up to 23.14% and 23.62%, respectively, but it did not cause any significant effects on the growth of HeLa cells stably transfected with wild-type MRS or MRS-SD (Fig. 5F). A similar pattern of cell viability under ROS stress was also observed with H_2_O_2_ treatment (supplementary material Fig. S1B). Moreover, the terminal deoxynucleotidyl transferase dUTP nick end labeling (TUNEL) assay indicated that cells expressing wild-type MRS or MRS-SD were more resistant to apoptosis upon exposure to ROS stimuli than empty-vector- or MRS-SA-expressing cells (Fig. 5G). The cell-protective effect of wild-type MRS under ROS stress was significantly reduced by U0126, suggesting that ERK activation is required for the cell-protective function of MRS (Fig. 5H; supplementary material Fig. S3E). In addition, unlike MRS, LRS overexpression did not compensate for the reduced cell viability caused by ROS (supplementary material Fig. S3F). These results indicate that dual phosphorylation of MRS is crucial for cell protection against oxidative damage.

**Fig. 6.**
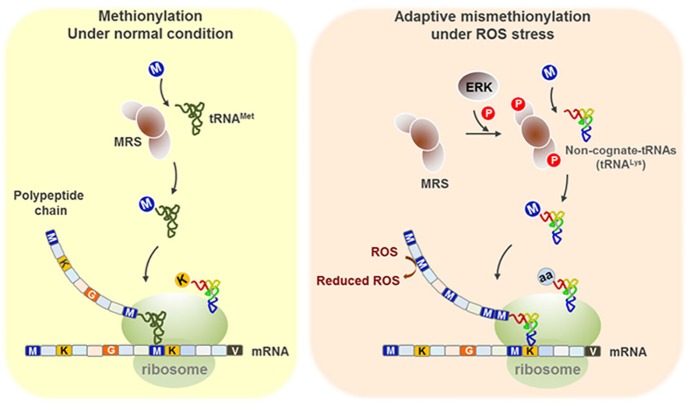
**Schematic model for the protective role of MRS under ROS stress.** Upon ROS stress, ERK is activated and phosphorylates MRS at the Ser209 and Ser825 residues. Phosphorylated MRS enhances the mischarging of Met on non-methionyl tRNAs, such as tRNA^Lys^. Met carried by non-cognate tRNAs is incorporated into growing polypeptides during translation and used as a ROS scavenger, protecting cells from oxidative damage and apoptosis.

In summary, under conditions of oxidative stress, MRS is phosphorylated at Ser209 and Ser825 by activated ERK. Dually phosphorylated MRS induces Met-misacylation of noncognate tRNAs. Increased numbers of Met residues resulting from misincorporation during translation can serve as ROS scavengers and protect cells from ROS-induced damage and apoptosis (Fig. 6).

## DISCUSSION

Previous studies have reported the importance of the Met residue as an antioxidant agent (Levine et al., 1996; Luo and Levine, 2009; Stadtman et al., 2002; Stadtman et al., 2003) and an increase in Met-misacylation and Met-misincorporation under ROS stress (Netzer et al., 2009). Although MRS mutation- or condition-dependent Met-misacylation has been identified in *Escherichia coli* and *Saccharomyces cerevisiae* (Jones et al., 2011; Wiltrout et al., 2012), the exact mechanism and meaning of this phenomenon in human cells is not yet understood. In this study, we identified human MRS as a new substrate for ERK during ROS stress and demonstrated a novel function of phosphorylated MRS – changing tRNA specificity to increase the rate of codon-independent Met-mistranslation during oxidative damage.

Accurate translation is an important issue for cells, allowing them to maintain their normal cellular integrity (Yadavalli and Ibba, 2012). To maintain translational fidelity, ARSs possess an editing function – they remove misactivated aminoacyl-adenylate or mischarged tRNAs by several mechanisms. Whereas other class Ia ARSs have at least one connecting peptide 1 (CP1) with an editing site and perform pre-transfer and post-transfer editing, MRS has a truncated CP1 domain and performs pre-transfer editing in a catalytic-site-dependent manner (Englisch et al., 1986; Fersht and Dingwall, 1979; Jakubowski, 1991; Yadavalli and Ibba, 2012). Although the manner in which human MRS performs its editing function and recognizes its cognate tRNA is structurally unclear, it is known that bacterial MRS recognizes the anticodon region and acceptor A73 in tRNA^Met^ (Senger et al., 1992). In eukaryotes, MRS is expected to have a certain additional mode for the recognition and aminoacylation of tRNA. First, the C-terminal WHEP (824–900 amino acids) domain strengthens the association of tRNA^Met^ with MRS under suboptimal tRNA concentrations (Kaminska et al., 2001). Second, the N-terminal 215–267 amino acids on the GST-like domain are crucial for MRS catalytic activity (He et al., 2009). Our results are in line with these studies in that phosphorylation in these appended domains, but not in the core domain, can modulate MRS enzymatic activity. The structural change in the MRS-SD mutant also supports the possibility of functional modulation by MRS modification (Fig. 2A). Although further investigation is required to understand the interaction of phosphorylated MRS with a non-cognate tRNA, it does not seem to recognize either the anticodon region or the discriminator base in the non-cognate tRNAs. The common bases between Met-misacylated tRNAs and tRNA^Met^ are mainly located in the D and TψC arms, implying that phosphorylated MRS might recognize tRNAs in a different way.

Wild-type MRS and the MRS-SD mutant showed similar levels of tRNAe^Met^ binding in EMSA, whereas their kinetic analyses suggested that MRS-SD charges tRNAe^Met^ less efficiently than wild-type MRS does (Table 1; Fig. 2B). The MRS-SD mutant, on the other hand, showed more effective aminoacylation activity to tRNA^Lys^. Considering only the Met-charging efficacy, the reduced susceptibility of phosphorylated MRS to tRNAe^Met^ is unnecessary. The reduced affinity to the cognate tRNAe^Met^ might be an inadvertent result of the increased selectivity for other tRNAs. The activity of MRS-SD for the initiator tRNA^Met^ (tRNAi^Met^), however, was similar to that of wild-type MRS, indicating that MRS phosphorylation under ROS stress might not cause adverse effects on translation initiation. Indeed, there was little difference between the overexpression of wild-type MRS and the MRS-SD mutant in their effect on global translation (data not shown).

Although MRS-SD revealed evident tRNA^Lys^-charging activity, MRS might have to compete with lysyl-tRNA synthetase (KRS) for capturing tRNA^Lys^ during ROS stress unless there is spare tRNA^Lys^. We analyzed free tRNA^Lys^ as well as charged tRNA^Lys^ using acidic urea PAGE gels and observed that some portion of free tRNA^Lys^ seems to be available even when KRS is fully active (data not shown). It also implies that other spare non-cognate tRNAs are available for misacylation. Met-misincorporation does not require high amounts of other tRNAs. It is known that the total increase in Met-misincorporation is about 10% under conditions of ROS stress (Netzer et al., 2009). Other possible mechanisms of increasing the availability of tRNAs during oxidative stress, such as changes in the accessibility of tRNAs to their cognate ARSs, cannot be excluded.

Considering that a series of tRNAs that mainly carry charged or polar amino acids are mismethionylated under ROS stress (Netzer et al., 2009), the shift in substrate preference can confer apparent benefits to cells, such as the localization of Met residues to the protein surface and an increased number of Met residues. According to Levine et al., not all of the Met residues in the original positions are used as ROS scavengers (Levine et al., 1996). Approximately 50% of the original Met residues in glutamine synthetase were oxidized by ROS, with the intact Met residues being buried within the core of protein. When repositioned in exposed spots with increased occupancy by misacylation, Met has a greater chance of reacting with ROS. In fact, the Met-misincorporated residues in FLAG-VN–AIMP3 were all detected on the surface of protein, supporting this idea (Fig. 4). Met-misincorporation increased the number of Met residues from the original 3 to 11 in VN (173 amino acids) and from 1 to 8 in AIMP3 (174 amino acids) (Table 2; Fig. 4).

Cells under normal conditions also had a basal level of Met-misincorporation on the protein surface (Fig. 3B; Fig. 4). This is probably due to endogenous ROS levels. Interestingly, the residues that were basally exchanged with Met did not perfectly match those detected following MRS-SD expression. This suggests that Met-misacylation by MRS can be arbitrary in some respects but can provide equal results under independent circumstances. This is probably due to the characteristics of non-cognate tRNAs, which can be used as substrates for phosphorylated MRS. Although the MRS-SD mutant showed extended affinity for a broad range of tRNAs, not all tRNAs were charged by MRS under ROS stress (Netzer et al., 2009). Generally, tRNAs carrying hydrophilic amino acids were preferentially used for Met-misacylation. Therefore, the selection of non-cognate tRNAs for Met-misacylation and coupled Met-incorporation seems to be regulated in a flexible way but within a limited range to cope with different environments.

Our finding that cells adopt a strategy involving MRS-mediated mistranslation to survive under ROS stress by tolerating a reduced fidelity of translation is unique. The sacrifice of translational fidelity does not seem to cause severe side effects in the short term, because cells transiently transfected with the MRS-SD mutant did not show any signs of apoptosis (Fig. 5B,D). Long-lasting misacylation, however, might cause adverse effects on cells due to the accumulation of misfolded or inactive proteins. Consistent with this expectation, stable cells expressing the MRS-SD mutant showed slightly reduced viability in the MTT assay (Fig. 5E). There might be a correlation between long-lasting misacylation and human diseases such as cancer or degenerative diseases, and this should be further studied to uncover their relationship. Nevertheless, the function of dually phosphorylated MRS during ROS stimulation is advantageous, at least for a short period, because it can induce Met-misacylation to remove ROS and to protect against protein damage while maintaining cell viability.

## MATERIALS AND METHODS

### Cell culture

HeLa and HEK293T cells were cultured in high-glucose Dulbecco's Modified Eagle's Medium (DMEM) supplemented with 10% fetal bovine serum (FBS) and 1% penicillin-streptomycin at 37°C under 5% CO_2_. To establish stably transfected HeLa cell lines, cells were transfected with the pcDNA3-Myc empty vector, wild-type pcDNA3-Myc-MRS, pcDNA3-Myc-MRS-SA or pcDNA3-Myc-MRS-SD plasmid using FuGENE HD (Roche). Stable cells were selected and maintained under antibiotic pressure (800 µg/ml of geneticin; Duchefa Biochemie).

### ROS induction and inhibitor treatment

Cells were cultured until they reached 80% confluence. Cells were treated with 4 µM sodium arsenite (Sigma) or 200 µM H_2_O_2_ for 4 h in DMEM with FBS (2% for short-term and 5% for long-term treatment) and 1% penicillin and streptomycin. Cells were pre-treated with the MAPK inhibitors SB203580 (p38 MAPK inhibitor), SP600125 (JNK inhibitor), and PD98059 or U0126 (ERK inhibitor) 1 h before ROS induction, at a concentration of 20 µM. All inhibitors were purchased from Calbiochem (Billerica). For the DPI chase (Enzo), cells were pre-treated with 50 µM DPI 30 min before ROS induction.

### DCFH-DA assay

HEK293T cells transfected with pcDNA3-Myc empty vector, wild-type pcDNA3-Myc-MRS, pcDNA3-Myc-MRS-SA, or pcDNA3-Myc-MRS-SD plasmids were exposed to sodium arsenite (4 µM) for 24 h. After treatment with 20 µM DCFH-DA (Invitrogen) (Ruiz-Ramos et al., 2009) for 15 min, cells were washed twice with PBS. The DCF signals were detected using a fluorescence microscope equipped with a green fluorescence filter (470 nm excitation, 525 nm emission) (Nikon) to monitor intracellular ROS levels.

### Immunoblotting

Cells were lysed in lysis buffer (50 mM Tris-HCl pH 7.4, 0.5% Triton X-100, 5 mM EDTA, 10% glycerol and 150 mM NaCl) containing phosphatase inhibitor and protease inhibitor (Calbiochem) for 30 min at 4°C. After centrifugation, supernatants were collected and the protein amounts were quantified by using the Bradford assay (BioRad). Proteins extracts from the cells were separated by SDS-PAGE, transferred to PVDF membrane and incubated with specific primary antibodies. Antibodies against phosphorylated (p)-Ser (Abcam), p-Thr (Cell Signaling Technology), p-Tyr (Cell Signaling Technology), Myc (Santa Cruz), FLAG (Sigma), MRS (Abcam), tubulin (Sigma), DsRed (Clontech), Bax (Santa Cruz) and Bcl-2 (Santa Cruz) were used in this study. Primary antibodies were used at a concentration of 0.2–0.4 µg/ml (Abcam, Sigma and Santa Cruz) or with a 1∶1000 dilution (Cell Signaling Technology).

### Immunoprecipitation

Protein extracts were incubated with primary antibody (2 µg) for 4 h at 4°C with agitation, and then incubated for a further 4 h at 4°C with Protein-A–agarose (Invitrogen). Beads were washed three times with cold lysis buffer and supernatants were removed. Samples were dissolved in the SDS sample buffer and separated by SDS-PAGE.

### 2D-PAGE

Protein extracts from HeLa cells were incubated with alkaline phosphatase (Roche) for 2 h. Each 500-µg protein extract was rehydrated in resolubilization buffer [7 M urea, 2 M thiourea, 2% ASB-14, 0.5% Triton X-100, 1% (v/v) ampholyte, 1% (v/v) tributylphosphine and 0.1% Bromophenol Blue]. Samples were loaded onto the immobilized pH gradient (IPG) strip gels (linear pH gradient 4–7, 7 cm; Bio-Rad) and subjected to isoelectric focusing (Bio-Rad).

### *In vitro* kinase assay and filter binding assay

GST-fusion MRS proteins were purified from *Escherichia coli* Rosetta 2. Protein expression was induced by treatment with 0.5 mM isopropyl-β-D-thiogalactopyranoside (IPTG), followed by culturing cells at 18°C overnight. Harvested cells were lysed by sonication, and lysates were incubated with glutathione Sepharose 4B (GE Healthcare) in lysis buffer (PBS containing 0.5% Triton X-100 and protease inhibitor) at 4°C for 6 h. Before the kinase reaction, the GST-fusion MRS proteins were pre-incubated with 500 µM ATP for 10 min at room temperature, and the kinase reactions were performed at 30°C for 30 min by adding ERK (Cell Science), [γ-^32^P]ATP (Izotop, SBP301, 1000 µCi) and kinase buffer (100 mM Tris-HCl pH 7.4, 75 mM MgCl_2_, 5 mM EGTA, 1 mM DTT, phosphatase inhibitor and protease inhibitor). Reactions were stopped by adding the SDS sample buffer and the samples were separated by SDS-PAGE and detected by autoradiography.

For the peptide kinase assay (Kwon et al., 2011), GFP–ERK expressed in HEK293T cells with or without U0126 pre-treatment was immunoprecipitated with the anti-GFP antibody. N-terminal biotinylated peptides were chemically synthesized (GL Biochem). Each peptide [3 mM MRS Ser209 (QKQPFQPSPAEGR), MRS S209A (QKQPQPAPAEGR), MRS Ser825 (GGQAKTSPKPA), MRS S825A (GGQAKTAPKPA), positive control (APRTPGGRR) and negative control (APRAPGGRR)] was allowed to react with the GFP–ERK at 30°C for 30 min. Each sample was filtered through a streptavidin-coated matrix biotin-capture membrane (Promega) using a 96-well Minifold filtration apparatus. The membrane was washed as described previously (Schaefer and Guimond, 1998) and exposed for autoradiography.

### Circular dichroism spectrum analysis

Wild-type MBP–MRS and MBP–MRS-SA and SD mutants were purified and eluted with a buffer containing 50 mM maltose at 4°C for 24 h and then dialyzed with 10 mM potassium phosphate buffer (pH 7.4). The circular dichroism spectrum was measured using a Jasco J-815 circular dichroism spectrometer at 25°C and 70°C in the far-UV range from 190 to 250 nm. Samples were loaded into a 1-mm path-length absorption microcell. The results are shown as an average of three repeated scans after subtraction of buffer background.

### Aminoacylation assay

His-tagged MRS (wild type, SA and SD) expressed in *E. coli* RIL strain was purified using ProBond Resin (Invitrogen) and washed with lysis buffer (pH 7.8, 20 mM KH_2_PO_4_, 500 mM NaCl, 10% glycerol and 2 mM 2-mecaptoethanol) changing the buffer pH from 7.8 to 6 to 5.2, and back to 6, with 24 mM imidazole at the final step. His–MRS was eluted in the presence of 300 mM imidazole (pH 6.0) and dialyzed with PBS containing 20% glycerol. tRNAe^Met^(CAU), tRNAi^Met^(CAU), tRNA^Lys^(CUU), tRNA^Ala^(AGC), tRNA^Gly^(GCC), tRNA^His^(GUG) and tRNA^Leu^(CAG) were synthesized by *in vitro* transcription. The MRS aminoacylation reaction was performed at 37°C in reaction buffer (30 mM HEPES, pH 7.4, 100 mM potassium acetate, 10 mM magnesium acetate, 4 mM ATP, 20 µM Met, 500 µg/ml of each tRNA, 400 nM purified MRS and 25 µCi [^35^S]Met (Izotop, 1000 Ci/mmol). For kinetics analysis, each tRNA (tRNAe^Met^, tRNAi^Met^ and tRNA^Lys^) was used at a concentration of 0.5 µM to 80 µM. Aminoacylation reaction samples were spotted on 3-mm filter paper pre-wetted with 5% trichloroacetic acid (TCA) containing 1 mM Met. Filter paper was washed three times with 5% TCA and dried, and radioactivity was detected by liquid scintillation counting (Wallac 1409).

### EMSA

tRNAe^Met^(CAU), tRNA^Lys^(CUU), tRNA^Ala^(AGC), tRNA^Gly^(GCC), tRNA^His^(GUG) and tRNA^Leu^(CAG) were synthesized by *in vitro* transcription with [α-^32^P]UTP (Izotop, 3000 Ci/mmol). Purified His-tagged MRS proteins (wild-type and MRS-SD; 0, 1, 2 and 2 µM) were mixed with each tRNA probe in the binding buffer (20 mM Tris-HCl pH 7.4, 75 mM KCl, 10 mM MgCl_2_ and 5% glycerol) and incubated at 30°C for 30 min. Samples were mixed with same volume of sample buffer and separated by 6.5% non-denaturing polyacrylamide gel electrophoresis. Radioactivity was detected by autoradiography.

### Mass spectrometry analysis

GST-fusion MRS domains (MD1 and MD3) were incubated with ERK and ATP, and were subjected to SDS-PAGE. MRS bands were cut from the SDS-PAGE gel and in-gel-digested with trypsin (Promega). Tryptic digests of MRS were subsequently separated by online reversed-phase chromatography for each run using a Thermo Scientific Eazynano LC II autosampler with a reversed-phase peptide trap EASY-Column (100-µm inner diameter, 2-cm length) and a reversed-phase analytical EASY-Column (75-µm inner diameter, 10-cm length, 3-µm particle size; both from Thermo Scientific), and electrospray ionization was subsequently performed using a 30-µm (inner diameter) nano-bore stainless steel online emitter (Thermo Scientific) and a voltage set at 2.6 V, at a flow rate of 300 nl/min. The chromatography system was coupled on-line with an LTQ VelosOrbitrap mass spectrometer equipped with an electron transfer dissociation (ETD) source. To improve peptide fragmentation of phosphopeptides, we applied a data-dependent neutral loss MS3 ETD mode or a data-dependent decision tree (DDDT) to select for collision-induced dissociation (CID) or ETD fragmentation, respectively, depending on the charge states. Protein identification was accomplished utilizing the Proteome Discoverer v1.3 database search engine (Thermo Scientific) and searches were performed against IPI.human.v3.2 FASTA database or human MRS FASTA database. A fragment mass tolerance of 1.2 Da, peptide mass tolerance of 15 ppm and maximum missed cleavage of 2 was set. Result filters was performed with peptide rank (maximum rank, 1), peptide number per protein (minimal number of peptides, 2; count only rank 1 peptides, true; count peptide only in top scored proteins, true) and charge state versus score [score to which the filter is applied, Sequest Node (XCorr); minimal score for charge state = +1: 1.7, +2: 2.5, +3: 3.2, >+4: 3.5]. The carbamidomethylation (+57.021 Da) of cysteine (C) was set as a static modification, and the following variable modifications were allowed; GlyGly/+114.043 Da (K), acetyl/+42.011 Da (K), hexNAc/+203.079 Da (N, S, T), phospho/+79.966 Da (S, T, Y), oxidation/+15.995 Da (M), deamidated/+0.984 Da (N, Q). All processed data were subsequently transformed to a .sf file with the Scaffold 3 program and, finally, all MRS post-translational modifications (PTMs) identified from control or stimulated sample were scored and compared with Scaffold PTM software.

For Met-misincorporation data analysis, protein identification was accomplished utilizing the Proteome Discoverer v1.3 database search engine (Thermo Scientific) and searches were performed against FLAG-VN–AIMP3 FASTA database. The carbamidomethylation (+57.021 Da) of cysteine (C) or deamidation (+0.984 Da) of asparagine or glutamine (N, Q) was set as a static modification or as a variable modification, respectively. To identify the exchange of specific amino acids to Met, the following variable modifications were additionally allowed; K→M/+2.946 Da (K), D→M/+16.014 Da (D), V→M/+31.972 Da (V), G→M/+74.019 Da (G), H→M/−6.018 Da (H), L→M/+17.956 Da (L), A→M/+60.003 Da (A) and oxidation/+15.995 Da (M). Finally, all Flag-VN–AIMP3 PTMs identified from both conditions were inspected manually.

### Met-misincorporation detection by radioisotope

HEK293T cells were transfected with pBiFC-VN173-AIMP3 and pcDNA3-Myc empty vector or pcDNA3-Myc-MRS (wild type, SA or SD) plasmids using Fugene HD transfection reagent (Roche). After 24 h, the cells were treated with 50 µM ALLN in methionine-free DMEM (Invitrogen) for 30 min. To confirm the effect of ERK on MRS-mediated mismethionylation, ERK inhibitor was added with ALLN. Then 1 mCi [^35^S]Met was added to the cells, followed by incubation for 2 h. Cells were further incubated with 4 µM sodium arsenite for 4 h, washed twice with PBS and then subjected to immunoprecipitation. After SDS-PAGE, the [^35^S]Met incorporation into the FLAG–AIMP3 protein was detected by autoradiography.

### TagRFP mutant generation and Met-misincorporation detection by fluorescence

PCR-based site-directed mutagenesis was performed to change Met67 in TagRFP (GenBank: BAI43881.1) to Lys (M67K), His (M67H) or Gly (M67G). Briefly, the *Hind*III fragment in pHAG016 into which TagRFP was cloned after the SRα promoter was swapped with *Hind*III digests of PCR fragments containing mutations in the 3′ PCR primer region.

Each TagRFP plasmid was co-transfected with pcDNA3-Myc empty vector or pcDNA3-Myc-MRS (wild type, SA or SD) into the HEK293 cells. After 24 h, the cells were treated with 4 µM arsenite and incubated for 4 h. Images were analyzed for snap pictures using MetaMorph software. The red fluorescent cells were counted using three randomly selected scopes in high-power fields (×20). Because of differences in the light absorption rate, light path and material properties of cell-culturing gels, the brightness is not uniform over the microscopic image. Non-uniform illumination correction is required as pre-processing before image analysis.

To analysis cell images based on fluorescence intensity, image contrast was adjusted using a localized version of the modification framework histogram smoothing (MF-HS) algorithm (Arici et al., 2009) to enhance the low-dynamic input image without producing over-enhancing artifacts in the resulting image. Then, the advanced simple linear iterative clustering (SLIC) superpixels method as used for cell segmentation. To identify the approximate boundary of cell, the degree of initial size and compactness parameters were used. The cell region of interest was manually selected after the SLIC superpixel method. The feature vectors were extracted from the selected cell object and used to analyze the differences in cell size and intensity.

### Cell growth and viability assay

Stably transfected HeLa cells expressing pcDNA3-Myc empty vector or pcDNA-Myc-MRS (wild type, SA or SD) were treated with 4 µM sodium arsenite and the cell growth curve was monitored at 37°C for 48 h using the IncuCyte Kinetic Live Cell Imaging System (Essen BioScience). To assess the cell viability, MTT (USB) stock solution (5 mg/ml) was added to a final concentration of 0.5 mg/ml in each well containing 200 µl of medium, followed by incubation for 30 min. The precipitated crystal was dissolved in 100 µl of DMSO (Sigma). Absorbance was measured at 450 nm using a microplate reader (Sunrise, Tecan).

### TUNEL assay

Stably transfected HeLa cells expressing pcDNA3-Myc empty vector or pcDNA-Myc-MRS (wild type, SA and SD) were treated with 4 µM sodium arsenite for 72 h and apoptosis was measured using Dead End Fluorometric TUNEL assay system (Promega,) according to the manufacturer's manual. ERK inhibitor was added 1 h before arsenite treatment when required.

### Statistical analysis

The statistical significance of data was determined by applying Student's *t*-test. Significance of analysis of variance is indicated in the figures as; *P<0.05, **P<0.01, ***P<0.001. Statistical analyses were performed with the Prism 5 software (GraphPad Software).

## Supplementary Material

Supplementary Material
