# Supplementary material for: Promiscuous methionyl-tRNA synthetase mediates adaptive mistranslation to protect cells against oxidative stress
Source: J Cell Sci. 2014 Oct 1;127(19):4234–45. doi: 10.1242/jcs.152470 (PMC4179492; doi:10.1242/jcs.152470)
Supplement: Supplementary Material [file supp_127_19_4234__index.html]

Supplementary Material 

# Promiscuous methionyl-tRNA synthetase mediates adaptive mistranslation to protect cells against oxidative stress

## JCS152470 Supplementary Material

**Files in this Data Supplement:**

- **Supplementary Material**
