# Supplementary material for: Promiscuous methionyl-tRNA synthetase mediates adaptive mistranslation to protect cells against oxidative stress
Source: J Cell Sci. 2014 Oct 1;127(19):4234–45. doi: 10.1242/jcs.152470 (PMC4179492; doi:10.1242/jcs.152470)
Supplement: Supplementary Material [file supp_jcs.152470_JCS152470.pdf]

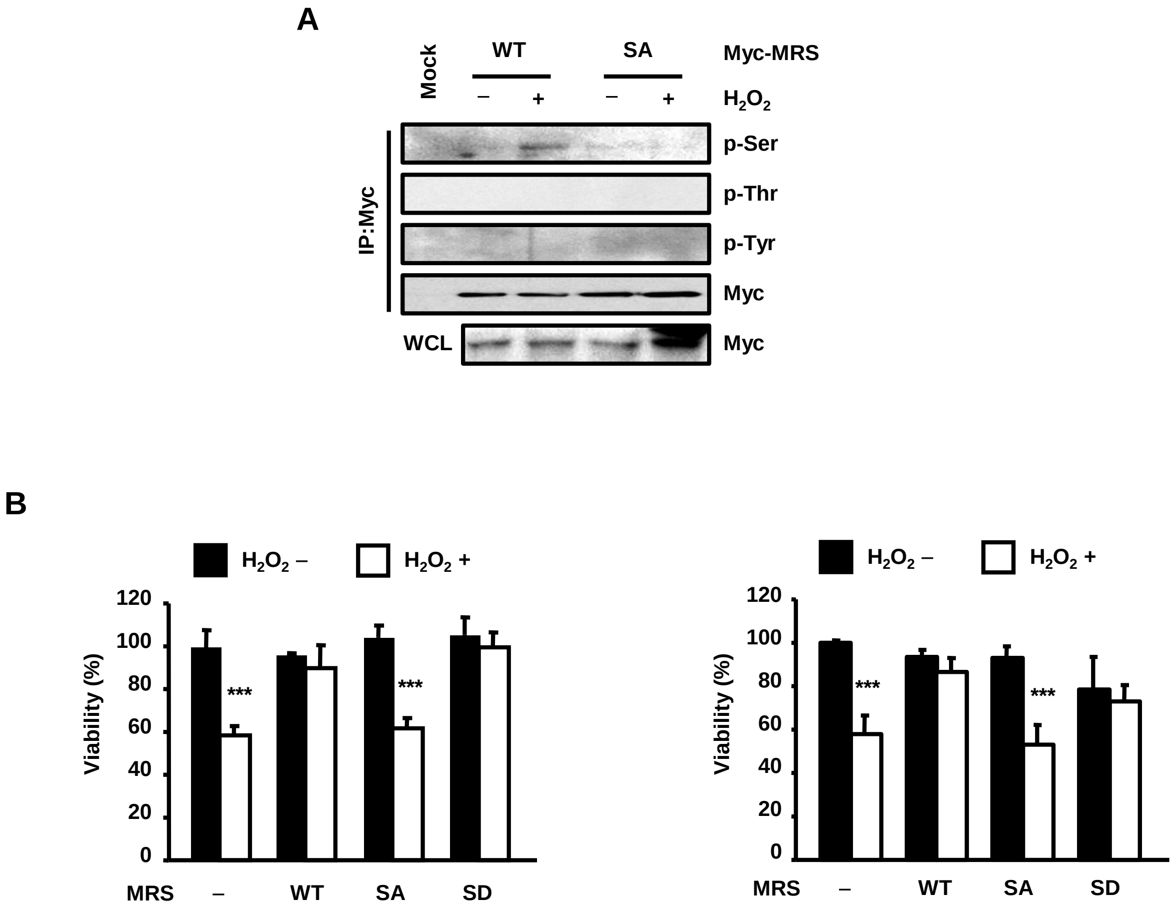

Fig. S1. MRS was phosphorylated and induced cell protection upon H_2O_2 stress. (A) HEK293T cells transfected with WT Myc-MRS or the Myc-MRS SA mutant were incubated with 200 μM H_2O_2 and the lysates were immunoprecipitated with anti-Myc antibody. H_2O_2-dependent phosphorylation of MRS was detected by p-Ser, p-Thr, and p-Tyr antibody. (B) The effect of WT MRS, the MRS SA or SD mutant on the cell viability under H_2O_2-induced ROS stress was determined by MTT assay. HEK293T cells transiently expressing each MRS protein (left) or HeLa cells stably expressing each MRS (right) were used for the viability analysis. The values of relative cell viability are represented as mean ± s.d. (n = 3). ***, P<0.001.

A

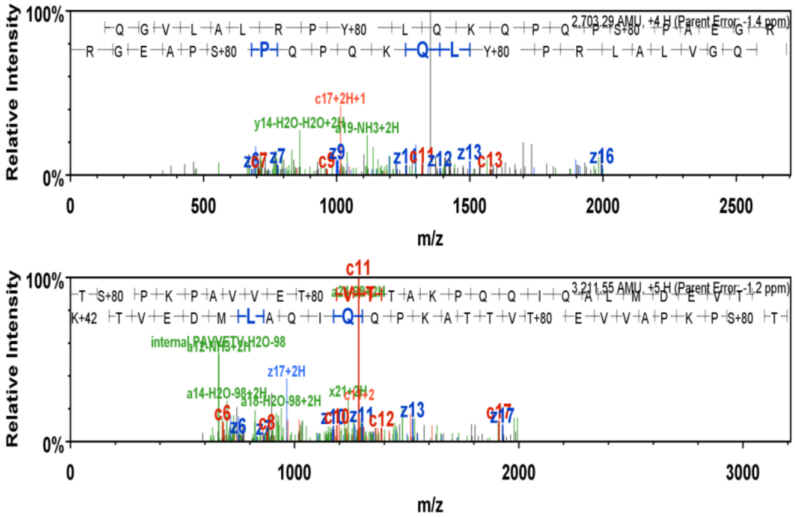

Domain	Sequence	Site
MD1	QGVLALRPyLQKQPQPsPAEGR	Y201, S209
MD3	TsPKPAVVEtVTTAKPQQIQALMDEVTK	S825, T833

B

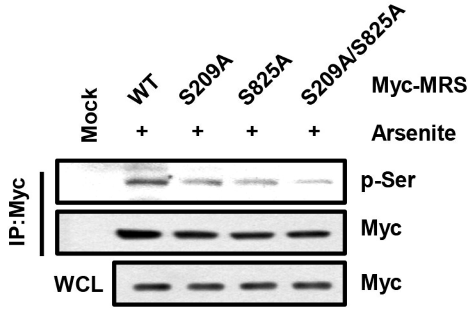

C

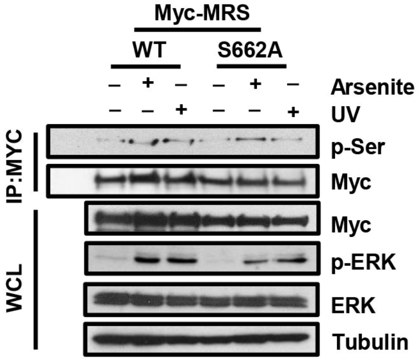

Fig. S2. Ser209 and Ser825 residues are the ERK-dependent phosphorylation sites in MRS under ROS stress. (A) GST-fused domains of MRS (MD1 and MD3), which were subjected to *in vitro* kinase reaction with purified active ERK, were processed for nano LC-MS/MS analysis as described in Materials and Methods. Peaks of peptides containing Tyr201 and Ser209 residues in MD1 (top) and Ser825 and Thr833 residues in MD3 (middle) are shown. The peptide sequences containing ERK-mediated phosphorylation were presented (bottom). (B) HEK293T cells transfected with WT Myc-MRS, Myc-MRS S209A, Myc-MRS S825A or Myc-MRS S209A/S825A mutant were treated with sodium arsenite. Cell lysates were immunoprecipitated with anti-Myc antibody and then immunoblotted with p-Ser antibody. (C) HEK293T cells were transfected with Myc-MRS WT and Myc-MRS S662A (GCN2 kinase phosphorylates S662 residue upon UV irradiation). Transfected cells were supposed to arsenite or UV (50 J/m^2) stress for 4 h or 30 min, respectively. Myc-MRSs were immunoprecipitated and then phosphorylation state was detected using p-Ser antibody.

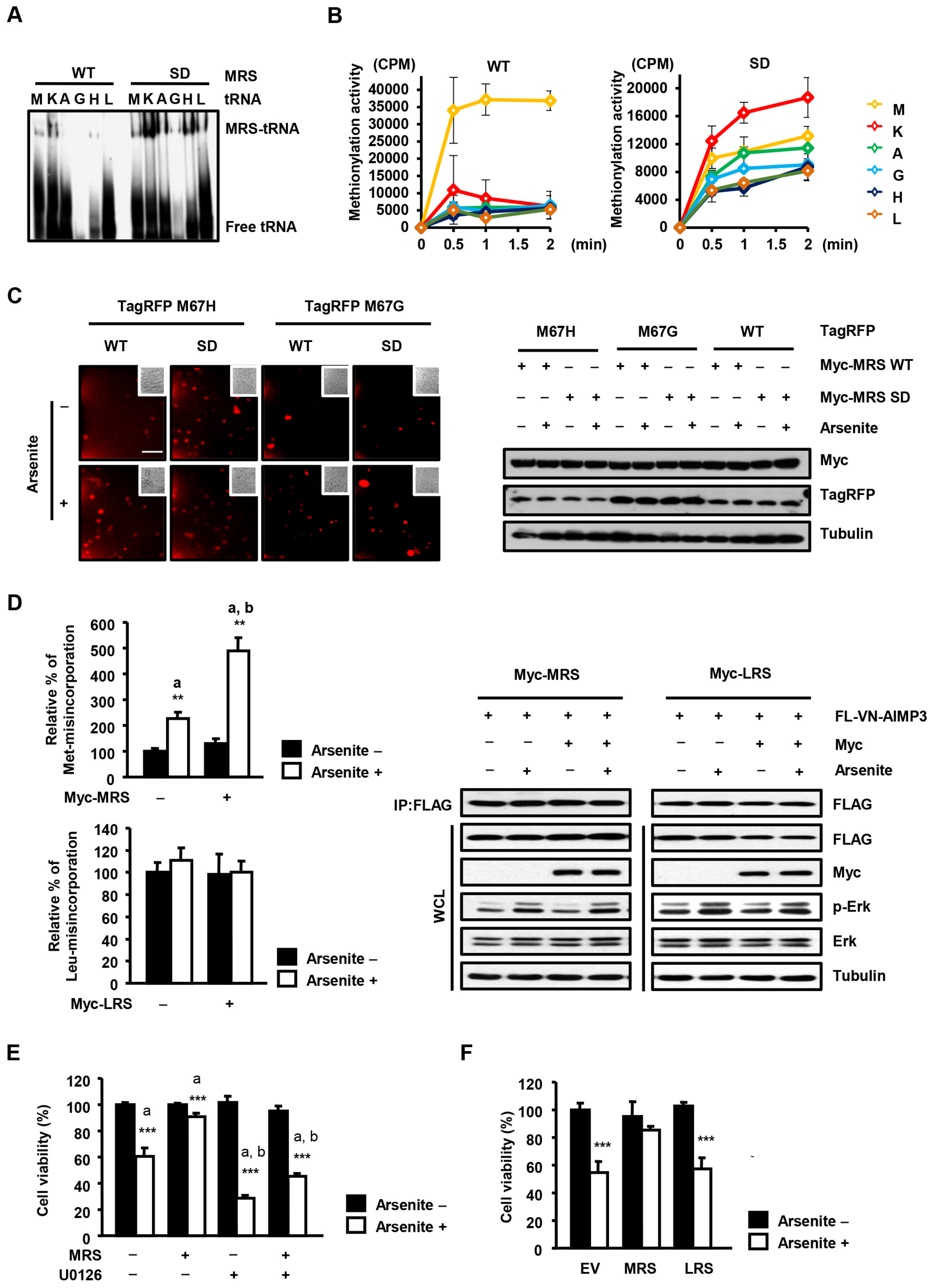
A
WT
SD
MRS
M K A G H L
M K A G H L
tRNA
MRS-tRNA
Free tRNA
B
(CPM)
WT
Methionylation activity
40000
35000
30000
25000
20000
15000
10000
5000
0
0 0.5 1 1.5 2 (min)
(CPM)
SD
20000
16000
12000
8000
4000
0
0 0.5 1 1.5 2 (min)
M
K
A
G
H
L
C
TagRFP M67H
TagRFP M67G
WT SD WT SD
Arsenite
–
+
M67H
M67G
WT
TagRFP
Myc-MRS WT
Myc-MRS SD
Arsenite
Myc
TagRFP
Tubulin
D
Relative % of Met-misincorporation
600
500
400
300
200
100
0
a, b
**
a
**
Arsenite –
Arsenite +
Myc-MRS – +
Relative % of Leu-misincorporation
140
120
100
80
60
40
20
0
Arsenite –
Arsenite +
Myc-LRS – +
Myc-MRS
Myc-LRS
FL-VN-AIMP3
Myc
Arsenite
IP:FLAG
FLAG
WCL
FLAG
Myc
p-Erk
Erk
Tubulin
E
Cell viability (%)
120
100
80
60
40
20
0
a
***
a
***
a, b
***
a, b
***
Arsenite –
Arsenite +
MRS – + – +
U0126 – – + +
F
Cell viability (%)
120
100
80
60
40
20
0
***
***
Arsenite –
Arsenite +
EV MRS LRS

Fig. S3. The MRS SD mutant charges Met to non-cognate tRNAs and Met-misincorporation is specific to MRS and ERK. (A) Binding affinity of WT MRS and the MRS SD mutant to several radioactively labeled tRNAs such as $tRNA_e^{Met}$(CAU), $tRNA^{Lys}$(CUU), $tRNA^{Ala}$(AGC), $tRNA^{Gly}$(GCC), $tRNA^{His}$(GUG) and $tRNA^{Leu}$(CAG) was determined by EMSA. Each tRNA probe was represented as M, K, A, G, H and L, respectively. (B) Methionylation activities of WT MRS (left) and the MRS SD mutant (right) to the several tRNAs as described above were compared by aminoacylation assay. The CPM (count per minute) values of methionylation to these tRNAs are represented as mean ± s.d. (n = 3). (C) HEK293T cells co-transfected with TagRFP M67H mutant (Met to His substitution) and Myc-MRS (WT or SD mutant) were treated with sodium arsenite. The red fluorescence increased by Met-misincorporation to the H67 residue was observed by fluorescence microscopy (×200). Met-misincorporation to G67 was also monitored in the TagRFP M67G mutant (Met to Gly substitution)-transfected cells. Insets show the same field as in the phase-contrast image. Scale bar = 80 μm (left). The expression level of Myc-tagged MRS proteins and TagRFP mutants were analyzed by immunoblotting (right). (D) HEK293T cells co-transfected with Flag-VN-AIMP3 and MRS were incubated with $[^{35}S]$Met in the presence with arsenite (left, top). For the cells transfected with Flag-VN-AIMP3 and leucyl–tRNA synthetase (LRS) together, $[^{3}H]$Leu was added instead of $[^{35}S]$Met (left, bottom). The cell extracts were immunoprecipitated with anti-Flag antibody. $[^{35}S]$Met and $[^{3}H]$Leu signals from the Flag-VN-AIMP3 were detected using liquid scintillation counter. The amounts of immunoprecipitated Flag-VN-AIMP3 and transfected Myc-MRS or Myc-LRS were analyzed using immunoblotting (right). **, P<0.01. a, P value indicates a significant difference between the arsenite-untreated and -treated groups; b, P value indicates a significant difference between arsenite-treated EV and MRS groups. (E) HEK293T cells transfected with EV or Myc-MRS were treated with arsenite. To inhibit ERK activation, cells were pretreated with U0126 1 h before being exposed to arsenite. After 72 h incubation, cell viability was analyzed using MTT assay. ***, P<0.001. a, P value indicates a significant difference between the arsenite-untreated and -treated groups; b, P value indicates a significant difference between arsenite-treated U0126 – and U0126 + groups. (F) HEK293T cells were transfected with EV, Myc-MRS and Myc-LRS, and the overexpression effect of these ARSs on cell viability under ROS stress was analyzed after 72 h incubation. ***, P<0.001.

FLAG-Venus N-term

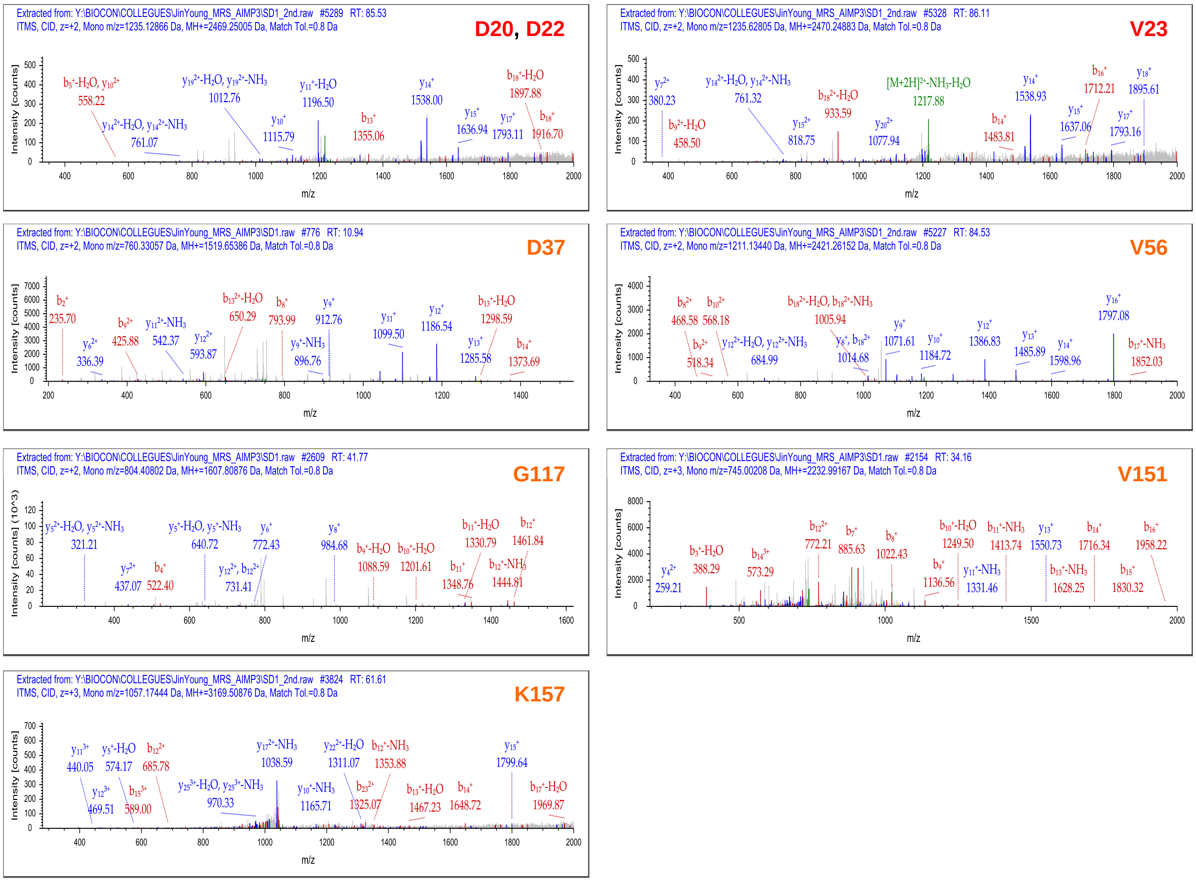

AIMP3

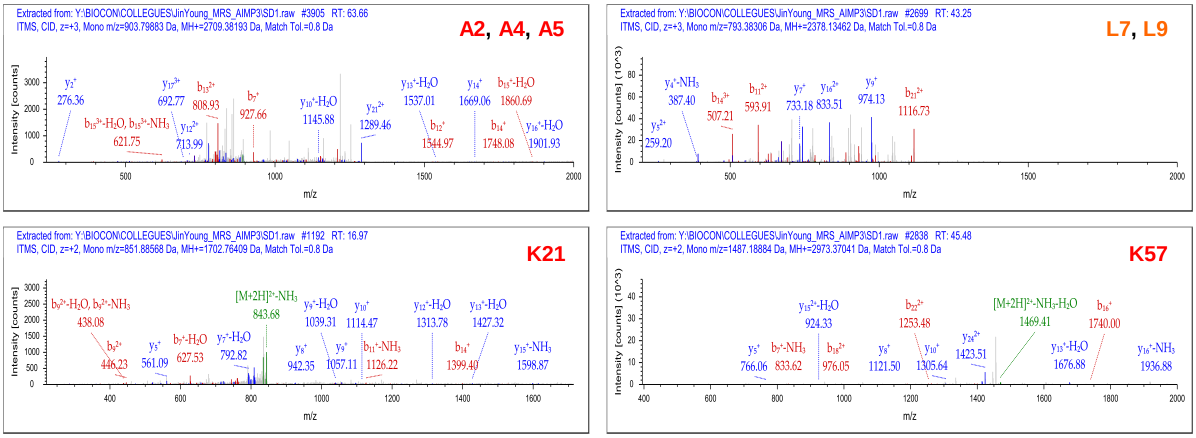

Fig. S4. Met-misincorporated residues in Flag-VN-AIMP3. Peptides from Flag-VN-AIMP3, which contain Met-exchanged residues were identified via mass spectrometry analysis. Peaks of peptides containing Met in the non-Met residues are shown.
